# Association of Low Vitamin D Status With Adiponectin and Fibroblast Growth Factor-21 in Newly Diagnosed Type 2 Diabetes Mellitus Patients

**DOI:** 10.7759/cureus.71448

**Published:** 2024-10-14

**Authors:** Visakamutharasi Murugiah, Pravati Pal, Jayaprakash Sahoo, Nivedita Nanda, Suryanarayana B Shamanna

**Affiliations:** 1 Physiology, Jawaharlal Institute of Postgraduate Medical Education and Research, Puducherry, IND; 2 Endocrinology, Jawaharlal Institute of Postgraduate Medical Education and Research, Puducherry, IND; 3 Biochemistry, Jawaharlal Institute of Postgraduate Medical Education and Research, Puducherry, IND; 4 General Medicine, Jawaharlal Institute of Postgraduate Medical Education and Research, Puducherry, IND

**Keywords:** adiponectin, fibroblast growth factor-21, interleukin-6, malondialdehyde, newly diagnosed t2dm

## Abstract

Introduction: Adiponectin and fibroblast growth factor-21 (FGF-21) have important roles in metabolic regulation and insulin sensitivity. Vitamin D is linked to metabolic dysregulation, inflammation, and oxidative stress in chronic type 2 diabetes mellitus (T2DM). The present study aimed to assess the relationship of vitamin D levels with adiponectin and FGF-21 in newly diagnosed T2DM.

Methods: A descriptive study was conducted on 47 patients with newly diagnosed T2DM recruited based on the American Diabetes Association criteria. Fasting plasma glucose and insulin were measured, and the homeostatic model assessment for insulin resistance was calculated. The serum levels of metabolic regulators such as vitamin D, adiponectin, and FGF-21; inflammatory markers such as interleukin-6 (IL-6), tumor necrosis factor-α, and high-sensitivity C-reactive protein; and oxidative stress markers such as malondialdehyde (MDA) and total antioxidant status were measured.

Results: The mean serum vitamin D concentration was 17.49±7.10 ng/ml, and all patients had low vitamin D levels (<30 ng/ml). Vitamin D was positively correlated with adiponectin (r=0.331, p=0.023), and it was negatively correlated with FGF-21 (r=-0.356, p=0.014), IL-6 (r=-0.411, p=0.004), and MDA (r=-0.515, p<0.001).

Conclusion: There was a reduction in vitamin D concentration in all subjects. Vitamin D showed a positive association with adiponectin and a negative association with FGF-21 and inflammatory and oxidative stress markers. Vitamin D deficiency might increase the risk of progression of T2DM in these subjects.

## Introduction

Type 2 diabetes mellitus (T2DM), characterized by hyperglycemia and insulin resistance, is a growing global issue, disrupting metabolic homeostasis and significantly impacting cardiovascular health. The prevalence of T2DM is expected to reach 134 million by 2045 in India [1]. Adiponectin and fibroblast growth factor-21 (FGF-21) are metabolic regulators with anti-diabetic action. Clinical research shows that low adiponectin levels are often observed even at the initial stage of T2DM, such as at the time of diagnosis [2]. Higher circulating adiponectin levels are reported to be associated with a lower risk of T2DM, even after adjusting for various influencing factors [3]. Animal models of T2DM have reported that the glucose transport from plasma to adipocytes is mediated by FGF-21, which is helpful in the normalization of plasma glucose levels in T2DM [4]. People with newly diagnosed T2DM [5,6] and long duration of T2DM have shown high serum FGF-21 levels, possibly suggesting deregulation in its production or impaired signaling in target tissues in response to high blood glucose levels [6]. A study has reported elevated serum FGF-21 levels in response to diabetes-induced early-stage atherosclerosis [5]. Along with metabolic dysregulation, elevated inflammatory and oxidative stress markers are observed in younger age group people with T2DM [7,8]. High serum levels of inflammatory markers such as interleukin-6 (IL-6), high-sensitivity C-reactive protein (Hs-CRP), and tumor necrosis factor alpha (TNF-α) are observed in people with T2DM [9]. Significantly high levels of malondialdehyde (MDA), with low total antioxidant capacity (TAC), are seen in newly diagnosed T2DM patients [8].

The hormone vitamin D can alter the insulin levels either through its direct action on pancreatic β-cells or indirectly through calcium regulation [10]. Vitamin D plays a pivotal role in alleviating inflammation, a significant factor in the development of insulin resistance. Additionally, vitamin D regulates the optimal level of reactive oxygen species in pancreatic β-cells, which tend to be elevated during diabetes. In cases of vitamin D deficiency, these vital processes deteriorate, leading to the progression of diabetes [11]. Serum 25-hydroxy vitamin D concentrations are used to assess vitamin D status, which should be maintained at levels above 30 ng/ml [12]. Vitamin D levels are decreased in newly diagnosed T2DM [7,8]. Vitamin D may be considered as an adiponectin secretagogue in diabetics [13]; however, no significant association of vitamin D deficiency with adiponectin was observed in patients with T2DM [14]. Clinical trials on the effect of vitamin D supplementation on adiponectin levels in diabetics have produced mixed results, with some studies showing increased adiponectin and improved metabolic markers, while others find no significant changes [15,16]. While vitamin D affects various metabolic pathways through its impact on insulin sensitivity and inflammation, FGF-21 plays a central role in managing glucose and lipid metabolism [17]. The association of vitamin D deficiency with FGF-21 in T2DM was not widely studied. Studies have shown that vitamin D deficiency is associated with elevated levels of CRP, IL-6, TNF-α, and MDA and lower levels of TAC, in newly diagnosed T2DM [7,8]. A prospective study in T2DM has established a significant negative association between vitamin D and TNF-α, but no significant association between vitamin D and Hs-CRP [18]. This evidence suggests that vitamin D deficiency may contribute to metabolic dysregulation, inflammation, and oxidative stress in T2DM patients.

However, there exists a paucity in studies that specifically analyzes the link of vitamin D with adiponectin and FGF-21 in individuals newly diagnosed with T2DM. As vitamin D has an active role in the pathogenesis of diabetes and its complications, in the present study, we assessed the link of vitamin D levels with adiponectin, FGF-21, and inflammatory and oxidative stress markers in newly diagnosed T2DM.

## Materials and methods

Study population

The present descriptive study was conducted at the Department of Physiology in collaboration with the Departments of Endocrinology, Biochemistry, and Medicine of Jawaharlal Institute of Postgraduate Medical Education and Research (JIPMER) in Puducherry, India. Forty-seven untreated newly diagnosed T2DM patients of either gender between the age group of 18 and 45 years, with 23 males and 24 females, attending the Medicine/Endocrinology Outpatient Department in JIPMER and staff and relatives of the staff of JIPMER comprised the study population. Diagnosis of diabetes was performed based on the American Diabetes Association criteria [19]. According to the criteria, subjects with fasting plasma glucose (FPG) ≥126 mg/dl are included in the study. The patients included in the study were neither taking any medicines to control blood glucose levels nor taking vitamin or antioxidant supplements. Patients with endocrine diseases other than T2DM, high blood sugar levels (≥200 mg/dl), complications, which require immediate blood sugar control, and a history of smoking and alcoholism were excluded. The study was approved by the Institute Ethics Committee for Human Studies of Jawaharlal Institute of Postgraduate Medical Education and Research (approval number: JIP/IEC/2018/470), dated December 27, 2018, and was done as per guidelines from the Declaration of Helsinki and Indian Council of Medical Research (ICMR). Written informed consent was obtained from all participants after explaining the details of the procedure in the patient's language.

Assessment of anthropometric parameters

Height was measured to the nearest centimeter using a stadiometer (VM Electronics Hardware Ltd, Chicago, IL, USA), which was mounted on a wall. Weight was measured using a weighing machine (Charder Electronic Co., Ltd, Taichung, Taiwan) to the nearest kilogram (kg). Body mass index (BMI) was calculated using Quetelet's index (BMI=weight (kilogram)/(height (meter))2). Asian criteria for BMI were followed [20].

Measurement of biochemical markers

Five milliliters of fasting venous blood samples from the antecubital vein was collected. The serum was separated and stored at −80°C for biomarker assessment. The serum level of glucose was measured immediately after blood collection using commercially available kits adapted to a clinical chemistry autoanalyzer based on the principle of spectrophotometry (Beckman Coulter AU680, Beckman Coulter, Inc., Brea, CA, USA) using the glucose oxidase-peroxidase method. Vitamin D levels were analyzed using the Siemens ADVIA Centaur XP analyzer (Munich, Germany) using the chemiluminescence assay technique. The serum levels of insulin, adiponectin, FGF-21, IL-6, TNF-α, Hs-CRP, and MDA markers were analyzed using a ChroMate semi-automated enzyme-linked immunosorbent assay (ELISA) analyzer. The serum level of total antioxidant status (TAOS) was measured with the colorimetric assay kit (ferric reducing antioxidant power (FRAP) method). The homeostatic model assessment for insulin resistance (HOMA-IR) index was obtained using the following formula: serum insulin (μIU/ml)×FPG (mg/dl)/405. The procedure was performed using standard operating protocols and following the guidelines of individual assay kits.

Sample size calculation

The required sample size was calculated as 47 using the statistical formula for descriptive study estimating a mean, http://www.sample-size.net/sample-size-conf-interval-mean/. The sample size was estimated at a 95% confidence level and a 5% precision, with the standard deviation of adiponectin as 8.7.

Statistical methods

The normality of the continuous data was assessed using the Kolmogorov-Smirnov test. Normally distributed data were represented as mean and standard deviation. Non-normally distributed data were represented as median and range. The correlation between the variables was analyzed using Pearson's correlation test for parametric data and Spearman's rank correlation test for non-parametric data. All statistical analyses were carried out for two-tailed significance, and a p-value of <0.05 was considered as statistically significant. Statistical analysis was performed using IBM SPSS Statistics for Windows, V. 21.0 (IBM Corp., Armonk, NY, USA).

## Results

We recruited newly diagnosed T2DM patients (n=47) of either gender between the age group of 18 and 45 years who were not under treatment. Patients' mean age at diagnosis and mean BMI were 39±4 years and 24±3 kg/m^2^, respectively. The distribution of baseline parameters is shown in Table 1. Table 2 shows the correlation between vitamin D and other biomarkers in newly diagnosed T2DM patients (n=47). Vitamin D was positively correlated with adiponectin (r=0.331, p=0.023). Vitamin D was found to be negatively correlated with FGF-21 (r=-0.356, p=0.014), IL-6 (r=-0.411, p=0.004), and MDA (r=-0.515, p<0.001).

**Table 1:** Distribution of baseline parameters in newly diagnosed T2DM patients (n=47) Data expressed as mean±SD and # median (range) for non-parametric data. T2DM: type 2 diabetes mellitus; BMI: body mass index; FPG: fasting plasma glucose; HOMA-IR: homeostatic model assessment for insulin resistance; FGF-21: fibroblast growth factor-21; IL-6: interleukin 6; TNF-α: tumor necrosis factor alpha; Hs-CRP: high-sensitivity C-reactive protein; MDA: malondialdehyde; TAOS: total antioxidant status

S. no.	Parameters	Newly diagnosed T2DM patients (n=47)
1	Age (years)	38.98±4.13
2	Height (cm)	160.28±8.77
3	Weight (kg)	62.38±11.37
4	BMI (kg/m^2^)	24.14±2.97
5	Vitamin D (ng/ml)	17.496±7.105
6	FPG (mg/dl)	152.66±19.33
7	Insulin (µIU/ml)	28.77±19.33
8	HOMA-IR	10.77±7.31
9	Adiponectin (µg/ml)	8.04±5.02
10	FGF-21 (pg/ml)^#^	32.77 (3.05-71.73)
11	IL-6 (pg/ml)^#^	26.84 (16.03-69.72)
12	TNF-α (pg/ml)^#^	15.40 (12.50-19.00)
13	Hs-CRP (mg/L)^#^	10.73 (3.50-11.98)
14	MDA (nmol/ml)	8.80±1.75
15	TAOS (mM/L)	0.90±0.32
16	Redox ratio^#^	10.10 (7.73-13.68)

**Table 2:** Correlation analysis of vitamin D on biomarkers in newly diagnosed T2DM patients (n=47) Pearson's correlation test was used for parametric data, and # Spearman's correlation test was used for non-parametric data. A p-value of <0.05 was statistically considered significant. ***p<0.001; **p<0.01; *p<0.05. T2DM: type 2 diabetes mellitus; FPG: fasting plasma glucose; HOMA-IR: homeostatic model assessment for insulin resistance; FGF-21: fibroblast growth factor-21; IL-6: interleukin 6; TNF-α: tumor necrosis factor alpha; Hs-CRP: high-sensitivity C-reactive protein; MDA: malondialdehyde; TAOS: total antioxidant status

S. no.	Parameters	Newly diagnosed T2DM patients (n=47)	
		r	p
1	FPG (mg/dl)	-0.042	0.781
2	Insulin (µIU/ml)	-0.107	0.472
3	HOMA-IR	-0.125	0.401
4	Adiponectin (µg/ml)	0.331	0.023*
5	FGF-21 (pg/ml)^#^	-0.356	0.014*
6	IL-6 (pg/ml)^#^	-0.411	0.004**
7	TNF-α (pg/ml)^#^	-0.004	0.980
8	Hs-CRP (mg/L)^#^	-0.022	0.886
9	MDA (nmol/ml)	-0.515	<0.001***
10	TAOS (mM/L)	0.013	0.931
11	Redox ratio^#^	-0.217	0.143

## Discussion

The present study was conducted on 47 untreated newly diagnosed T2DM participants with a mean age of 39 years and a BMI of 24 kg/m^2^. The baseline FPG and insulin levels were elevated (normal FPG level ≤125 mg/dl and insulin level 2-25 µIU/ml), which was supported with high HOMA-IR (10.77±7.31) values. The HOMA-IR >1.9 indicates early insulin resistance, and values >2.9 indicate significant insulin resistance [21]. As the HOMA-IR values were quite high in these subjects, there was marked insulin resistance, though the plasma glucose level was not very high. Vitamin D was found to be lesser (17.496±7.105 ng/ml) than the normal range of 30-100 ng/ml in all the participants, and this finding is similar to that of Laway et al. [22]. In the present study, newly diagnosed T2DM patients had metabolic dysregulation along with vitamin D deficiency.

Hypovitaminosis is prevalent in India irrespective of age, physical state, and region, and in the adult age groups, the prevalence has been reported to be 30-91.2% [23]. The finding is similar to other studies on T2DM [22,24], where vitamin D deficiency was reported in recently established diabetic patients with an age of onset of <25 years [22], as well as in the >25-year age group with a T2DM duration of <6 months [22]. Sustaining the normal serum level of vitamin D has the advantage of reducing the risk of T2DM [12]. The pathogenesis of hypovitaminosis D-induced T2DM is possibly due to the hereditary variations in vitamin D receptors and vitamin D-binding protein genes, which lead to a state of glucose intolerance that subsequently alters the synthesis and secretion of insulin finally ending up in T2DM [10]. Adiponectin and FGF-21 are potent anti-diabetic hormones. Adiponectin executes its anti-diabetic action through the inhibition of insulin resistance. Adiponectin's role as a marker of insulin sensitivity makes it a valuable tool for assessing metabolic dysfunction early in the disease [25]. Vitamin D level was positively correlated with adiponectin (p=0.023). This finding suggests that adiponectin generation is dysregulated in T2DM and it is linked to the vitamin D level. FGF-21 promotes glucose utilization and energy expenditure by improving insulin sensitivity in the adipose tissue and stimulating thermogenesis in the brown adipose tissue. As a result, FGF-21 encourages the use of glucose for heat production rather than energy storage. Interestingly, despite its positive effects, FGF-21 levels are often elevated in states of insulin resistance and T2DM [17]. FGF-21 is an emerging therapeutic agent for T2DM [26], and the association of decreased vitamin D level with higher FGF-21 (p=0.014) indicates that vitamin D might be influencing the actions and regulation of FGF-21, which has a role in glucose homeostasis. Adiponectin and FGF-21 are powerful predictors of adverse cardiovascular events [27,28]. The association of vitamin D with adiponectin and FGF-21 has not been reported so far, and hence ours is the first report.

Vitamin D deficiency has been implicated in the pathophysiology of conditions associated with inflammation. It has been reported that vitamin D deficiency reduces the downregulation of nuclear factor-κB activity, subsequently leading to a pro-inflammatory state, with an increase in pro-inflammatory cytokines IL-6 and TNF-α and a decrease in anti-inflammatory cytokine IL-10 [29]. Along with the manifested inflammation in T2DM, oxidative stress-induced dysfunction of the endothelium makes the cells resistant to the hormone insulin and the eventual progression of T2DM and cardiovascular disease [30]. MDA is an oxidant that increases with an increase in the oxidative load of the body. With the increase in oxidant load, the antioxidant level decreases, as measured by TAOS. The redox ratio is the ratio of MDA and TAOS, and it indicates the oxidative stress level. In the present study, a high redox ratio indicates an increased oxidative stress in the subjects. Vitamin D is a potent antioxidant that regulates the production of glutathione, thereby alleviating oxidative stress and inflammation [31]. In the present study, vitamin D level was negatively correlated with inflammatory marker IL-6 (p=0.004) and oxidative stress marker MDA (p<0.001). The findings are similar to the reports of Kumar et al. [7,8]. This suggests that with the decrease in vitamin D level, oxidative stress and overall inflammatory state are enhanced in T2DM subjects. Vitamin D deficiency itself is a risk factor for cardiovascular disease [32]. The increased FGF-21 and reduced adiponectin level, along with oxidative stress and inflammation in vitamin D-deficient newly diagnosed T2DM subjects, could further increase the cardiovascular disease risk in them.

In summary, the findings of the present study suggest that reduced vitamin D level is associated with increased FGF-21 and reduced adiponectin level, inflammation, and oxidative stress in newly diagnosed T2DM subjects. This highlights the importance of screening for vitamin D deficiency in patients with newly diagnosed T2DM and taking appropriate corrective measures that may be useful to curb the progression of the disease and reduce the cardiovascular disease risk in them.

The present study did not include a longitudinal follow-up to determine the causality between vitamin D levels, insulin resistance, and cardiovascular risk factors over time. Although associations between vitamin D deficiency and metabolic dysregulation were established, the study did not explore the underlying mechanisms in detail. Further follow-up research is needed to elucidate the pathophysiological pathways involved in vitamin D's role in T2DM and associated cardiovascular risks. The present study does not account for confounding variables such as dietary habits, physical activity levels, socioeconomic factors, and genetic factors, which may influence vitamin D levels and metabolic health.

## Conclusions

Vitamin D deficiency is prevalent in newly diagnosed type 2 diabetics. With a decrease in vitamin D level, there are an increase in FGF-21 level, a reduction in adiponectin level, high oxidative stress, and inflammation in these subjects that may predispose them to increased cardiovascular disease risk. Being a descriptive study, the cause-effect relationship could not be established. Future prospective studies need to be conducted with a larger sample size to address this issue. Vitamin D deficiency is a treatable condition, which, when treated appropriately early in the course of the disease, may show improvement in glucose metabolism, inflammation, and oxidative stress states and may decrease the progression of the disease in newly diagnosed type 2 diabetics. Vitamin D supplementation might be an attractive therapy to regulate glucose metabolism in T2DM. Vitamin D can be suggested as an adjunct for newly diagnosed T2DM patients in addition to conventional therapy.
